# Life Satisfaction in Young Adults—The Moderating Role of Parental Support

**DOI:** 10.3390/ijerph191912513

**Published:** 2022-09-30

**Authors:** Shirley Ben-Shlomo, Noga Levin-Keini, Einat Ofir-Barash

**Affiliations:** 1 The Louis and Gabi Weisfeld School of Social Work, Bar Ilan University, Ramat Gan 52900, Israel; 2 The School of Social Work, Ashkelon Academic College, Ashkelon 78211, Israel

**Keywords:** emerging adulthood, family systems theory, life satisfaction, social support, parental support, personal resources, self-determination theory

## Abstract

The transition to adulthood in Western societies, termed emerging adulthood (EA), holds new challenges for family relationships across and within generations. Drawing on Self Determination Theory and Bowens’ Family systems theory, this study examines the combined contribution of personal resources and relationships with parents and friends to satisfaction with life among young Israeli adults. It also examines the possible change in parental support that occurs with increasing age. A convenience sample (*n* = 572) of young Jewish Israeli adults (18–29 years) completed structured questionnaires. Using regression analysis, we modelled the associations between personal and support variables to life satisfaction as well as the interactions between age and parental support. The findings reveal that young women reported receiving more parental support than young men. Commitment to values and beliefs and greater support from friends make a positive contribution to life satisfaction. High parental support impairs life satisfaction as age increases. The study extends Bowens’ theory to understand the developmental stage of young adulthood and emphasize on the practical level that therapists need to familiarize themselves with the protective variables at this stage of life and the changing role of parental support.

## 1. Introduction

The transition to adulthood in Western societies, termed emerging adulthood (EA), holds new challenges for family relationships across and within generations [1]. Until about thirty years ago, it was commonly believed that parental support reached its climax in adolescence, in parallel with the process of identity formation [2]. The understanding that this transition may be a lengthier process, occurring at ages 18–29 [3], has changed both research and therapeutic views on the journey to adulthood. This issue has become more significant since the outbreak of COVID 19, which has created changes in the family structure and employment status of young adults around the world [4].

One of the key understandings in this regard is that relationships with parents continue to be significant even beyond adolescence [5] and that parent–child interaction and experiences with friends, along with personal resources, may contribute to the young adult’s satisfaction with life, which is an important aspect of adjustment [6]. However, despite the clinical understanding about the integrated role of the individual’s relationships, alongside their personal resources, only a few studies have been conducted to date with young adults in order to identify the main determinants of life satisfaction in this stage of life [7]. Moreover, to the best of our knowledge, these variables have not been examined in the context of young adults in Israel, who are the focus of the current study.

Given the cultural context in which young adults live, they are critically impacted by the manner in which they experience their transition to adulthood and by the results of this transition [8]. Israel embodies individualistic and collectivistic values existing in tandem. On the one hand, it is considered a developed, Western country that emphasizes the needs of the individual. At the same time, the country holds dear collectivistic values, attaching great importance to family and community. This duality appears to be the product of Israel’s history and its establishment in the aftermath of the Holocaust [8]. Further, young israeli adults of both genders are required to serve in the army for a period of two to three years, providing them with an experience that is significant in terms of their future [9].

Our study is aimed at examining the combined contribution of personal resources (traumatic-stressful life events, self-mastery, identity formation) and relationship variables (financial and emotional support by parents and social support from friends) to satisfaction with life among young Israeli adults. In order to enable a more in-depth look at the relationship between young adults and their parents we sought to examine not only the nature of the association between their relationship and life satisfaction but also the change in the need for parental support as the young adult advances in age, a question that to the best of our knowledge has not been examined to date.

This question was raised in reference to Arnett’s theory [10], according to which young adults’ development stage may be conceived as an “in-between age”, during which they assume responsibilities and commitments but at the same time fail to perceive themselves as mature adults, while remaining dependent on their parents. This perception is challenged by social expectations of them: a previous study found that when young adults feel that they are not fulfilling society’s expectations, as commensurate with their age, their life satisfaction declines [11]. The hypotheses and questions formulated in the present framework were derived from two theories—Self-determination theory (SDT) [12,13], which emphasizes the contribution of personal and environmental resources to fulfilling the individual’s basic psychological needs and enhancing his life satisfaction; and Bowen’s family systems theory, which explains how potent relational forces ensure survival and facilitate less anxious physiological states that are crucial to wellness [14,15]. Life satisfaction is one of the most well-established indicators of general wellness and positive functioning [16], especially among young adults [9]. The concept refers to an individual’s cognitive assessment of how satisfied they are with their life in terms of the criteria they set themselves [16]. Some studies indicate that EA is characterized by an increase in satisfaction with life [17,18] and a decrease in negative affect in the form of depression and anxiety [1,10]. In contrast, others report a rise in mental problems [11,19]. 

A research conducted in 90 different countries also found gender differences in this regard, with women tending to have a higher subjective well-being compared to men [20]. Past research indicated that young women rely more on support from their environment than young men [21]. One explanation for this focuses on different cultural expectations from young men, who are perceived as being able to fend for themselves at this stage of life—unlike women, who are assumed to be supported by their partners [22].

The search for personal and supportive factors that may protect young adults at this developmental stage ties in with the claim of Bowen [14] that the individual’s developmental processes occur between two counterbalancing agents: togetherness and individuality [15]. Togetherness refers to the person’s ability to be in a meaningful relationship with others. Individuality represents the person’s ability to retain a coherent sense of self and a clear identity, even given his connections with others.

Bowen posited that functional families have a higher capacity for containing the individual’s separation, while continuing to provide support and approval [23]. Therefore, the young adult’s supportive relationships at this stage in life are of great significance, although importance is also attached to personal resources [6]. A number of studies on young adults revealed a developmental advantage for those with stronger personality resources [10,17]. The importance of these variables is emphasized, especially for those with previous exposure to traumatic-stressful life events, such as accidents, violence and terror attacks; these could lead to a developmental deficit and a more intense experience of developmental stress [24,25], thereby reducing satisfaction with life [26]. 

### 1.1. Personal and Relationship Resources

Bowen [14] claimed that the ability to be flexible and act wisely, even in situations of anxiety and stress, is associated with an individual’s personal and relationship resources [14]. The instability that characterizes EA may be mitigated by a strong sense of self-mastery, which may serve as a protective factor [17,27]. Self-mastery, one of the personal resources on which we chose to focus, is defined as the individual’s sense that they can influence circumstances and events in their life [28]. According to the theory of self-determination It refers to a feeling of competence, and a desire to be effective and in control of the development process using new skills [29]. A high sense of self-mastery at this stage of life was found to be associated with less emotional distress and greater life satisfaction [30].

One of the individual’s most significant psychological needs according to the theory of self-determination is the need for autonomy, namely, the desire to feel intrinsically free to act and make one’s own decisions [12]. This need is in keeping with the second variable we chose—Identity formation. Marcia [31] claimed that the status of identity achievement is measured by the individual’s position with respect to two dimensions: exploration and commitment. Exploration relates to the degree to which young adults consider the direction their life should take, while exploring different roles and commitments—this being a measure of the degree to which they are committed to their choices, or the extent to which their life choices reflect their values and beliefs [32]. As regards commitment, the findings of a cross-cultural study indicate that well-being is consistently associated with high commitment, high in-depth exploration, and low reconsideration of commitment [33].

The third psychological need to which the theory of self-determination refers is relatedness. Meaning the need to belong and be part of a group [34].

In the framework of the young adult’s relationships, we chose to focus on two aspects that are perceived as important at this developmental stage: parents [35,36], and friends [11]. Parents can influence their offspring’s life satisfaction through various types of support [37], including financial support and different levels of emotional support, such as advice, comforting and listening [36,38]. Many young adults feel that their parents are too involved in their lives, whether they live at home or elsewhere [10]. Nevertheless, the literature contains conflicting findings regarding the contribution of the family in general, and parents in particular, to young adults’ development [10,39]. According to studies in Southern European countries, young adults report that they feel satisfied with their dependence on emotional and financial support from their parents [40], although, to the best of our knowledge, the differential contribution of each type of support to the life satisfaction of young adults has not yet been measured. The present study is intended not only to answer this lacuna, but also to understand in greater depth whether the importance of parental support changes as a function of age, i.e., whether advancing age in the young adults affects their need for parental emotional and/or financial support.

In this regard a study encompassing five counties in southeastern Pennsylvania and four counties in New Jersey reported a correlation between intensive parental support and better psychological adjustment [38], Another study in which the majority of youngsters identified their ethnicity as European American/White found that when young adults are at an age where they are expected to live independently, and that expectation was not met, they reported lower well-being [11].Moreover, in light of the findings that social relations and friendship networks are a major source of satisfaction with life [41,42], together with parental support [43,44], the current study also focused on the support that participants received from their social networks. The importance of support from friends among young adults is discussed extensively in the literature [11,45]. For example: lack of social support among university students was found to be associated with increased stress [46] and more suicidal ideation [47]. In the present study, as stated above, our aim was to expand the understanding regarding the combined contribution of support from parents and friends.

### 1.2. The Current Study

In light of the literature review, we hypothesized that differences in life satisfaction will be found between men and women, with women being more satisfied with life than men (Hypothesis 1). We also hypothesized that differences will be found between men and women in the level of support relationships, with women relying more on parents’ emotional and financial support, as well as social support from friends, than men (Hypothesis 2). 

Two research questions were formulated: (1) What is the unique contribution of personal resources (traumatic-stressful life events, self-mastery and identity formation), together with financial and emotional support from parents and supportive relations with friends, to the explained variance in young adults’ satisfaction with life? and (2) What is the moderating role of financial and emotional parental support in the relationship between young adult’s age and satisfaction with life?

## 2. Method

### 2.1. Participants and Procedure

Our study was conducted in 2018. Approvals were obtained from the ethics committee of Bar-Ilan University (022102). The procedures used in this study adhere to the tenets of the Declaration of Helsinki. A total sample size of 98 participants was needed, as determined by G*Power for this study design with six predictor variables (setting the a priori effect size of 0.15, p-level to 0.05, power to 0.80). As we wanted to add background variables as covariates and explore possible interactions, and assuming 10% attrition, it was determined that a minimum of 150 participants was needed.

A total of 600 young Jewish Israeli adults received a set of structured questionnaires as well as written information about the study through social networks. No remuneration was offered for their participation. After eliminating incomplete questionnaires and those that did not meet the inclusion criteria (18–29 years of age and able to understand and complete questionnaires in Hebrew), a final sample was selected, consisting of 572 participants—326 (57%) women and 246 (43%) men. The participant’s gender was determined by the gender identity according to which the participant chose to present himself or herself.

The mean age for males and females was 25.66 (SD = 0.16) and 25.59 (SD = 0.17), respectively. The majority of participants were Israeli-born (87.8%), single (62.9%), employed (64.1%), and self-defined secular (64.1%). Forty-four (7.7%) were parents. A total of 62.7% rated their economic status as average (the average income in Israel is NIS 10,428, equivalent to £2200 or $3000).

### 2.2. Measures

**The Satisfaction with Life Scale** (SWLS) [16], assesses the individual’s global perception of their life satisfaction. The scale consists of 5 items (e.g., “So far I have gained the important things I want in life”). The participants were asked to indicate the degree to which they agree with each statement on a scale ranging from 1 (strongly disagree) to 7 (strongly agree). Each participant was assigned a score equal to the sum of their responses, with higher scores indicating greater life satisfaction. Cronbach’s α in the current study was 0.84.

**The Traumatic-Stressful Life Events Questionnaire** (TLEQ) [48] measures exposure to stressful life events. The 26 items in the questionnaire describe a variety of stressful events, such as road accidents, violence, etc. Included is an open-ended question, enabling the respondent to refer to an event not described in any of the other items. The participants were asked to indicate the number of times they had been exposed to each type of event, their age at the time, and the degree to which it had affected their lives on a scale of 1 (no effect on my life at all) to 5 (strongly affected my life). As there is no reason to believe that there is any connection between the items, it is accepted practice not to examine the reliability of the questionnaire [29]. 

**The Self-Mastery Scale** [49], assesses the individual’s sense of control over their life in the present and future, as well as over their environment. The scale consists of 7 items (e.g., “What happens to me in the future mostly depends on me”). The participants were asked to indicate the degree to which they agreed with the statement in each item on a 7-point Likert scale from 1 (strongly disagree) to 7 (strongly agree). Each participant was assigned a score equal to the mean of their responses, with higher scores indicating a greater sense of self-mastery. Cronbach’s α in the current study was 0.80.

**The Social Support from Parents Tool** (SSPI) [50] is adapted from a scale designed to assess intergenerational relations from the standpoint of the children. The adapted version consists of 4 items assessing the frequency of two types of parental support: financial (1 item: “How often have your parents helped you financially in the past 12 months?”); and emotional (3 items, e.g., “How often have your parents comforted you in the past 12 months?”). As the tool relates to frequency rather than perception, it is an indirect measure of parental support. Participants are marked on a 5-point Likert scale from 1 (never) to 5 (always). The participants were assigned a score for each aspect of support that is equal to the sum of their responses to the relevant items, with higher scores indicating more financial or emotional support from parents. Cronbach’s α in the current study was 0.75 for the total questionnaire and 0.85 for emotional support. As financial support consisted of a single item, internal reliability could not be tested.

**Social Support from Friends Scale** (SSPF) [51]. The scale consists of 5 items and assesses support from friends (e.g., “I feel comfortable turning to my friends when I have a problem”). The participants were asked to indicate the degree to which they agreed with each statement, marking their responses on a 5-point Likert scale ranging from 0 (strongly disagree) to 4 (strongly agree). Each participant was assigned a score equal to the mean of their responses, with higher scores indicating the receipt of greater support from friends (Cronbach’s α = 0.91).

**Inventory of the Dimensions of Emerging Adulthood—Revised** (IDEA-R) [52], This consists of 25 items assessing the two dimensions of identity: exploration and commitment. Five items relate to creating commitment (e.g., “I know what I want to achieve in life”), 5 to identifying with the commitment (e.g., “My plans for the future give me a sense of confidence”), 5 to wide-ranging exploration (e.g., “I think about the direction I want my life to take”), 5 to in-depth exploration (e.g., “I think about the future plans I have made for myself”), and 5 to ruminative exploration (e.g., “I am always searching for my direction in life”). The participants were asked to indicate the degree to which each statement applied to them, marking their responses on a 5-point Likert scale from 1 (strongly disagree) to 5 (strongly agree). A reliability of 0.86 was found for the tool as a whole. Factor analysis revealed that two separate factors explained 34% and 15% of the variance: the first was composed of the items in creating commitment and identifying with the commitment; the second was composed of the items in wide-ranging exploration, in-depth exploration and ruminative exploration. Participants were therefore assigned two scores equal to the mean of their responses to the relevant items: commitment (Cronbach’s α = 0.87) and exploration (Cronbach’s α = 0.85).

### 2.3. Data Analysis

Analysis was conducted in three stages. First, *t*-tests were performed to examine the differences between men and women with respect to satisfaction with life and social support. In order to assess the size of the effect, use was made of the scale according to Cohen [53]. The range of values up to 0.2 was defined as small, between 0.2 and 0.5 as medium, and above that as large. Next, Pearson correlations were conducted between the independent variables and satisfaction with life. Correlations that were considered significant were those whose α value was lower than 0.05. In accordance with Cohen [53] we defined the size of r = 0.1 as small, r = 0.3 as medium and r = 0.5 A large.

Finally, regression analysis was performed to examine the unique and combined contribution of the independent variables to the prediction of satisfaction with life. Each stage was considered significant when the α value was lower than 0.05. The size of an additional effect was represented as *f*^2^. The scale of values was according to that of Cohen [16]: *f*^2^ = 0.2 was considered small, *f*^2^ = 0.15 was considered medium, and *f*^2^ = 0.35 was considered large. PROCESS procedure [54] was used to explore interactions between the variables. This approach examines correlations between the independent variable and the dependent variable for various moderator levels [54]. For the variable of age, use was made of the approach by Johnson & Neyman [55], which is based on the entire range of moderator values [22]; the approach examines significance in the slope of a large number of values, e.g., age throughout the range of ages. It is thus possible to identify the exact age at which there is a change in the relationship between the independent variable and the dependent variable under the influence of the moderator.

## 3. Results

The research hypothesis relating to differences between men and women in satisfaction with life were examined using independent samples *t*-tests. As can be seen in Table 1, according to the study’s first hypothesis, women reported more life satisfaction compared to men. In addition, and in accordance with the second hypothesis, women reported receiving more parental support of both types than men, although a significant difference was found only for emotional support. A significant difference between the genders was found for support from friends, with women reporting more support from friends than men.

Pearson correlations between personal and supportive variables and life satisfaction were examined as a preliminary step to regression analysis (Table 2).

In order to examine the first research question regarding the unique and combined contribution of the study variables to the explained variance of satisfaction with life, hierarchical regression was performed. The participants’ background variables were entered in Step 1. In addition to the participants’ age, gender, and stressful life events that were significant to the purposes of the current study, we also entered four control variables (education, financial status, religiosity, and parenting), which were found to be associated with life satisfaction in the preliminary analyzes. Self-mastery was entered in Step 2; the identity dimensions (commitment and exploration) in Step 3; the dimensions of parental support and support from friends in Step 4; and the interactions between age and parental support (financial and emotional) in Step 5. The results appear in Table 3.

The study variables explained a total of 42% of the variance in life satisfaction. The background variables in Step 1 accounted for 9% of the variance, with higher education, greater religiosity, and parenthood associated with higher life satisfaction, and more past stressful life events associated with lower life satisfaction. The size of the effect was found to be small to medium.

Self-mastery in Step 2 contributed 21% to the explained variance, indicating that a greater sense of self-mastery is associated with higher life satisfaction. The size of the effect for this additional step was medium to large.

In Step 3 the identity dimensions accounted for a further 6% of the variance, with commitment positively and significantly contributing to the young adult’s life satisfaction, i.e., those who felt they lived in accordance with their principles and preferences reported higher life satisfaction. No significant contribution was found for exploration. The size of the effect for this addition was small to medium.

The support variables entered in Step 4 added another 2% to the explained variance, with greater support from friends associated with higher life satisfaction. No association was found between either type of parental support and life satisfaction. The size of the effect for this addition was small.

Step 5 examined the interactions between age and parental support, which accounted for 4% of the variance in life satisfaction. The size of the effect for the additional interactions was large.

**Figure 1:**
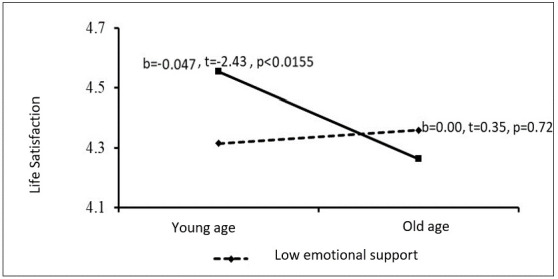
Emotional parental support as a moderating variable between age and life satisfaction.

The PROCESS procedure was performed to learn the source of this interaction, and the Johnson and Neyman [55] approach was used to examine the significance of the slope at a large number of age cut-offs as dependent on the moderating variable of parental support (financial and emotional). The results revealed that at low levels of emotional parental support, age was not associated with life satisfaction (*b* = 0.00, *t* = 0.35, *p* = 0.72). However, when parental emotional support was higher, age was negatively associated with life satisfaction (*b* = −0.04, *t* = −2.24, *p* < 0.01). In other words, among young adults who receive considerable emotional support from their parents, life satisfaction declines the older they get (Figure 1).

**Figure 2:**
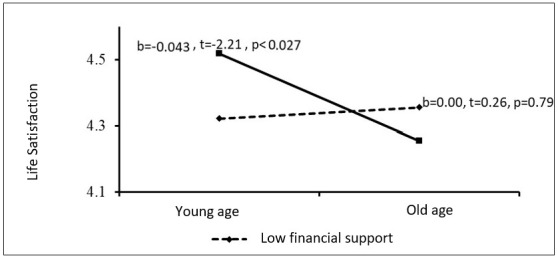
Financial parental support as a moderating variable between age and life satisfaction.

The same pattern was found for financial support. There was no association between age and life satisfaction at low levels of financial parental support (b = 0.00, t = 0.26, *p* = 0.79), but life satisfaction declined with age among individuals receiving more financial parental support (b = −0.04, t = −2.21, *p* < 0.02). Thus, as young adults get older, the more they rely on their parents for emotional and financial support, the less satisfied they are with their lives (Figure 2).

## 4. Discussion

The current study, based on self-determination theory (SDT) [39,56] and Bowen’s family systems theory [6]. It examined the contribution of young adults’ personal resources and relationships with parents and friends to life satisfaction. The study also examined the interaction between the young adults’ age, parental support and social support on the one hand, and life satisfaction on the other.

In keeping with the research hypothesis, we found gender differences both in life satisfaction and in the use of support systems. In line with previous studies [20,21] young women were more satisfied with life than men, and relied more on emotional support from both their parents and their friends. This finding marked gender as a protective factor in this period of life. possibly associated with the common perception of masculine culture, whereby men are expected to be assertive, competitive and focused on material success. Women, in contrast, are expected to be nurturing and focused on people and quality of life. A research study carried out by Cleary [57] found in this regard that while young men in distress desperately sought closer social relationships and support from family and friends, they feared being judged as emotionally vulnerable, feeble and emasculated, deterring them from actively seeking support and rendering them more prone to suicide.

Although we did not formulate specific hypotheses regarding the participants’ background variables, the results suggested that greater religiosity, a higher education, and parenthood were all protective factors for young Israeli men and women in EA, contributing to higher satisfaction with life. Religion has been found in the past to reduce depression and increase pleasure and satisfaction with life among young adults [58,59]. The findings are in line with self-determination theory, according to which religiosity contributes to the fulfillment of fundamental psychological needs, leading to a higher sense of mental well-being [60].

The association between a higher education and life satisfaction is also in line with past studies, which argued that academic achievements lead to higher satisfaction with life because the hope and motivation to succeed that are associated with the acquisition of an education mitigate concrete difficulties [56].

In the present study, youngsters’ parenthood was also associated with a greater satisfaction with life. This finding may be seen in light of Arnett’s [1] depiction of EA as a “role-less role” that generates feelings of anxiety, confusion, and a lack of belonging [1]. Moreover, the transition to parenthood could provide a social framework [61] in a period lacking in stable frameworks of this sort.

Our results indicated, in keeping with previous studies [26], that young Israeli adults who experienced past stressful life events reported lower satisfaction with life. At the same time, the findings indicated that higher self-mastery contributed significantly to higher satisfaction with life. A number of studies on young adults suggested a developmental advantage for those with stronger personality resources [10,17,62]. The needs emphasized by self-determination theory—competence, autonomy and relatedness—were found in the present research to be important for satisfaction with life on the part of young adults.

Self-mastery was associated with the assessment of challenges as being less threatening as well as with higher life satisfaction [63] and, as such, may serve as a protective variable against traumatic events experienced in the past. We also found, in line with a previous study, a contribution of the identity dimension of commitment to life satisfaction [55]. This means that the set of values and beliefs with which the individual identifies and is committed to could serve as a stable base in the unstable EA period [3]. Exploration as a dimension of identity was not found to be associated with satisfaction with life in the current study. Since exploration lies at the core of identity formation, being an experiential process that enables the young adult to probe different domains and directions for life [64], it is recommended to further examine this variable in future studies.

As mentioned, supportive relationships with both parents and friends are considered a crucial factor in satisfaction with life at this age, and are essential for emotional achievements in psychological development throughout life [26]. The above support systems reinforce the sense of belonging on the part of the individual and contribute, according to self-determination theory, to satisfaction with life [34].

Although numerous studies have explored the association between parental support and offspring achievements and adaptation [39,65], only a few relate specifically to satisfaction with life [21,66], and even fewer to the type of parental support [38]. In the present study, a positive, albeit weak, association was found between parents’ emotional support and satisfaction with life. Previous studies have found that relationships perceived by young adults to detract from their satisfaction with life are characterized by excessive involvement or interference on the part of the parents [65,66]. Relationships perceived to contribute to satisfaction with life are described as supportive of autonomy [67]. 

In order to gain a deeper understanding of these findings, and based on the second research questions, we examined whether parental support of the young adult moderated the association between age and life satisfaction. Interestingly, among young adults reporting low levels of parental support, age was not associated with life satisfaction. However, with respect to both financial and emotional support, among those reporting average levels of support or above, older age was associated with lower life satisfaction. To understand why parental support detracts from satisfaction with life as the young adult ages, reference may be made to Winnicott’s [68] theory of the “good enough mother” who adapts her parenting to the offspring’s abilities. As soon as the child is capable of fulfilling his or her own needs and can deal with frustration, the mother loosens her hold accordingly. One of the mechanisms that enable young adults to achieve autonomy is flexible parental interference, and its decline over time [69]. The older the young adult, the greater the expectation, both from themselves and from society, that they live an independent life. Emotional, and primarily financial, reliance on the parents may therefore be regarded as a sign of immaturity [11].

In contrast to parental support, support from friends was found in the present study to contribute directly to satisfaction with life. Young adults spend a great deal of time with their friends, and generally report a decline in the frequency of their interactions with their parents [70]. In adolescence, relationship needs begin to be met by friends rather than parents, and this process continues into EA [70]. Support from friends also has a mitigating effect on stress, neutralizing the influence of stressful events and the pressures of day-to-day life typical of this age [70].

## 5. Limitations

Several limitations of the current study should be noted. First, only self-report tools were used. Although this is common in such studies, the empirical picture they produce may not be complete. Secondly, the data was collected largely through social media, so that the sample consisted of young Israeli adults who are active in internet forums and therefore cannot be considered as representative. In addition, data was collected at a single time point. The associations described here could change over time, particularly in view of the important role determined for age. Finally, the study relates to young adults in Israel as a homogeneous group, without reference to subpopulations that make up the multicultural country [71]. If the concept of life satisfaction is seen as socially constructed, this would indicate that its constituents are always dependent on historical, collectivistic and social factors. The history of Israeli society has to a great extent honed the concept of family ties and family support. Despite being a Western society, Israel incorporates both individualistic and collectivistic values [72], and it would be interesting to examine if the connections found in the present study are true of other cultures as well.

## 6. Conclusions

This study expands on self-determination theory [12] and Bowen’s family systems theory [14] for understanding the developmental stage of young adulthood. Although the study was conducted before the global crisis created by the COVID 19 epidemic, the fact cannot be ignored that one of the consequences of the crisis was changes in the family unit and employment of many young adults. The findings of the study may be of great significance for understanding the variables that could help increase the satisfaction of these young adults with their lives. Based on self-determination theory, the study highlights the importance of the combined role of parental support and support from friends as well as the young adult’s personal resources in the context of satisfaction with life [73,74]. The study sheds light on a range of variables allied with social roles, including gender, education, religiosity and parenthood, that constitute protective factors against EA as a period characterized by instability.

The gender differences found in the study suggest the importance of developing differential interventions for men and women in EA. The manner in which each of the genders experiences reliance on parental support and support from friends appears to differ, and this should be taken into account both at the research and therapeutic levels.

Furthermore, planning interventions with young adults should address those who have experienced past stressful life events and who are especially vulnerable to mental distress during this period [73]. These youngsters may benefit from the existence of high self-mastery and commitment: the association between commitment and life satisfaction not only marks this variable as a protective factor but also emphasizes how it can help in commitment to the values of Israeli society, which stress the importance of family.

Emotional support from parents and support from friends were also found to be protective factors. The finding that the relationship between support and life satisfaction is a function of age highlights for therapists the importance of relating to the age of the young adult. In this context, the present study points to the importance of social relations as a source of support in such a developmental period, with the trusted basis for these relations moving from the parents to the friends.

## Figures and Tables

**Table 1:** Means, Standard Deviations, and *t*-tests for the Study Variables in the Two Study Groups.

	Women *n* = 321		Men *n* = 251			
	M	SD	M	SD	t (df = 570)	Cohen’s d
Satisfaction with Life	4.47	1.09	4.28	1.14	2.00 *	0.17
Emotional parental support	3.25	1.06	2.8	1.01	5.06 ***	0.43
Financial parental support	3.2	1.4	3.03	1.48	1.43	
Stressful life events	2.73	0.24	2.78	2.28	0.24	
Self-mastery	2.97	0.19	2.98	0.51	0.19	
Commitment	3.42	0.67	3.48	0.77	0.99	
Exploration	2.7	0.25	2.7	0.28	0.02	
Support from friends	3.38	0.53	3.2	0.62	3.54 ***	0.31

* *p* < 0.05. *** *p* < 0.001.

**Table 2:** Pearson Correlations between Independent Variables and Satisfaction with Life.

Satisfaction with Life	
Age Gender Education Financial status Religiosity Parenthood Stressful life events	0.02 0.03 0.13 ** 0.07 0.20 ** 0.16 ** −0.15 ***
Self-mastery	0.46 ***
Commitment	0.46 ***
Exploration	0.01
Emotional parental support	0.10 *
Financial parental support	0.00
Support from friends	0.28 **

* *p* < 0.05. ** *p <* 0.01. *** *p* < 0.001.

**Table 3:** Hierarchical Regression Coefficients (Beta Weights) for Satisfaction with Life.

	β	t	f^2^	∆R^2^
**Step 1**			0.1	0.09 ***
Age	0.04	0.82		
Gender	0.03	0.66		
Education	0.12	2.44 **		
Financial status	0.06	1.49		
Religiosity	0.15	3.54 ***		
Parenthood	0.11	2.63 **		
Stressful life events	−0.14	3.31 **		
**Step 2**			0.27	0.21 ***
Self- mastery	0.46	12.95 ***		
**Step 3**			0.06	0.06 ***
Commitment Exploration	0.28 0.05	7.36 *** 1.57		
**Step 4**			0.02	0.02 ***
Emotional parental support	0.04	1.13		
Financial parental support	0.02	0.49		
**Step 5**			0.72	0.04 ***
Age × Emotional parental support	0.02	2.28 *		
Age × Financial parental support	0.02	1.93 *		
*R* ^2^				0.42 ***
*F(13,557*)				14.27 ***

* *p* < 0.05; ** *p* < 0.01; *** *p* < 0.001.

## Data Availability

The data presented in this study are available on request from the corresponding author.
